# Construction of predictive models for contralateral occult thyroid carcinoma and central lymph node metastasis in unilateral papillary thyroid carcinoma using machine learning

**DOI:** 10.3389/fonc.2025.1623075

**Published:** 2025-09-05

**Authors:** Yaqi Zhao, Chunping Liu

**Affiliations:** Department of Breast and Thyroid Surgery, Union Hospital, Tongji Medical College, Huazhong University of Science and Technology, Wuhan, China

**Keywords:** thyroid neoplasms, occult neoplasms, lymphatic metastasis, thyroidectomy, machine learning

## Abstract

**Background:**

This study aimed to develop predictive models based on preoperative clinicopathological and imaging features to accurately assess the individual risk of contralateral occult thyroid carcinoma (OTC) and determine the number of central lymph node metastasis (CLNM) in patients with unilateral papillary thyroid carcinoma, thereby providing actionable guidance for surgical planning.

**Methods:**

Seven widely-used machine learning algorithms were employed to develop predictive models. Hyperparameter tuning was performed via cross-validation in combination with grid search. The models were subsequently trained and evaluated by using the optimal hyperparameter combinations. To facilitate comparative analysis, ROC curves, calibration curves were generated and DCA was performed. The optimal model was then selected on the basis of this comprehensive evaluation. Furthermore, a clinical prediction model was constructed utilizing the significant predictors identified.

**Results:**

The logistic regression model was identified to be the optimal predictive model. For the clinical prediction model of OTC, the following independent variables were incorporated: body mass index, and ultrasonographic findings, including capsular disruption, number of malignant nodules within a unilateral lobe, sum of the longest diameter (SLD) of tumors, and the presence of isthmic malignant nodule(s). This model yielded an area under the ROC curve (AUC) of 0.74 and 0.70 in the training and validation cohorts, respectively. For the clinical prediction model of ≥5 CLNM, the incorporated independent variables included: age, sex, chronic lymphocytic thyroiditis, and ultrasonographic features covering malignant nodules located near the isthmus, SLD, capsular disruption, and calcification. This model produced an AUC of 0.75 and 0.71 in the training and validation cohorts, respectively. Decision curve analysis indicated that clinical interventions guided by the two models could provide net benefit within threshold probability ranges of 10% to 90% and 10% to 70% for patients with PTC. And the calibration curves demonstrated a good agreement between model predictions and actual observations.

**Conclusion:**

This study developed and validated clinical prediction models to estimate the risk of contralateral OTC and the presence of ≥5 CLNM in patients with unilateral PTC. These models were designed to prevent overtreatment in low-risk patients while providing evidence-based guidance for decision-making about treatment choice in high-risk patients.

## Introduction

Thyroid carcinoma (TC) represents a malignancy of the endocrine system originating from thyroid follicular epithelial cells or parafollicular (C) cells (1) and its incidence has been on the rise rapidly across the globe. Reports from the National Central Cancer Registry of China indicated that the annual incidence of TC is rising at a rate of roughly 20%, making it the fourth most common malignancy among urban females (2). This upward trend may be partially attributed to advancements in diagnostic imaging techniques and fine-needle aspiration biopsy (FNAB), coupled with the implementation of public health screening programs (3). While previous studies have implicated genetic predisposition and environmental factors in TC pathogenesis (4), mounting evidence suggests a broader spectrum of potential risk factors. These factors involve unhealthy lifestyle habits, overweight/obesity, chronic psychological stress, environmental pollution, radiation exposure, and comorbid chronic conditions (5). Furthermore, specific risks for TC risk in females encompass a history of abortion, irregular menstruation, oral contraceptive use, and changes in estrogen and progesterone levels (6). Histologically, TC has distinct subtypes in terms of histopathological features and cellular differentiation. Among these, papillary thyroid carcinoma (PTC), a subtype of differentiated thyroid cancer (DTC) with a favorable prognosis, is most prevalent, accounting for approximately 85-90% of all cases (7). Consequently, its disease-specific mortality rate has remained relatively stable over the past three decades.

Initial surgical management constitutes the most critical phase in the treatment for PTC patients. Complete resection of the primary tumor and potentially involved adjacent tissues, combined with systematic lymph node dissection, significantly reduces the risk of locoregional recurrence and distant metastasis while providing essential data for accurate clinical staging and risk stratification (8). While overdiagnosis of thyroid cancer might lead to unnecessary treatments and healthcare resource consumption (9), it is crucial to acknowledge that not all micro-PTCs are behaviorally indolent. Notably, a subset of PTCs, even those measuring only a few millimeters, can develop extensive lymph node metastasis or cause significant local invasion, adversely impacting patient quality of life and survival. For a minority of patients harboring aggressive tumors, early and definitive intervention remains necessary. Consequently, the 2023 Chinese Guidelines for the Diagnosis and Treatment of Thyroid Nodules and Differentiated Thyroid Cancer unequivocally designate surgical resection as the standard treatment. Therefore, determining the optimal extent of surgery is paramount in clinical decision-making concerning PTC treatment. Clinical guidelines for PTC management are currently well-established. Unilateral lobectomy plus isthmusectomy has become the standard approach for unifocal DTCs less than 1 cm without high-risk factors (10). Current guidelines recommend total or near-total thyroidectomy for the following scenarios: tumor diameter > 4 cm, positive resection margin, extrathyroidal extension (ETE), vascular invasion, clinical lymph node metastasis (≥5 nodes or diameter ≥3 cm), or distant metastasis. However, debate still lingers about the optimal surgical management for PTC tumors measuring 1–4 cm in diameter, with studies yielding conflicting results regarding the impact of different surgical extents on prognosis (11). Meanwhile, definitive surgical recommendations for patients with unilateral multifocal carcinoma lack a consensus across guidelines. Cervical lymph nodes, particularly central nodes, are the most frequent site of metastasis in PTC (12) and constitute an independent risk factor for recurrence and diminished survival (13). Prior studies indicated that approximately 95% of recurrent or metastatic DTC occurred within the neck (14), with metastases to cervical or mediastinal lymph nodes accounting for 74% of these recurrences (15). Therefore, the assessment of lymph node metastasis status is paramount for surgical decision-making, estimation of recurrence risk, and selection of subsequent treatments.

Nevertheless, limitations persist with current guidelines. For instance, challenges remain when preoperatively determining critical indicators (such as PTC pathological subtypes, vascular invasion, and lymph node metastasis) that dictate surgical decision-making. In clinical practice, surgical planning relies heavily on preoperative imaging findings. Patients routinely undergo imaging evaluations, with the 2023 Chinese Guidelines recommending high-resolution ultrasonography as the primary imaging modality for thyroid nodule assessment. Ultrasonography effectively detects nodules >2 mm in diameter and characterizes them based on morphology, echogenicity, internal structure, and vascularity. It is highly sensitive in identifying the number or the size of the nodules, and malignancy-associated high-risk features (anteroposterior-to-transverse ratio, calcification, and capsular disruption) (13). However, ultrasonography has some inherent limitations. Results are operator-dependent and subject to interpretive subjectivity. Deep-seated lesions or those obscured by adjacent structures may evade detection. Furthermore, due to resolution constraints, nodules ≤2 mm carry a substantial risk of being missed (16). Consequently, patients whose preoperative ultrasonography reports unilateral malignancy may harbor pathologically confirmed OTC in the contralateral lobe. Ultrasonographic evaluation must also encompass cervical lymph nodes, but assessing the central lymph nodes is notoriously challenging: lymph nodes in this region are deeply located, exhibit complex and heterogeneous echotexture, and visualization is often difficult due to overlying thyroid tissue. The presence of micro-metastases further compromises the ultrasonographic accuracy in detecting CLNM (17). Therefore, surgeons should conduct an individualized risk-benefit assessment when facing ambivalence between total thyroidectomy (TT) and thyroid lobectomy (TL). For unilateral tumors, TL offers advantages in that it avoids overtreatment and carries lower complication risks. However, TL is not recommended for patients deemed at high risk of postoperative recurrence (18, 19). Salvage completion thyroidectomy following recurrence is significantly more complex and hazardous due to distorted anatomy and scar tissues, increasing the risks of recurrent laryngeal nerve injury and permanent hypoparathyroidism. Patients are also faced with additional anesthetic risks and higher medical expenditure. Conversely, for patients having undergone TT whose final pathology reveals neither contralateral OTC nor high-risk for nodal metastasis, TL could have been initially chosen based on patients’ choice, potentially preserving thyroid function.

This study aimed to develop clinical prediction models by utilizing preoperative data to guide surgical decision-making for unilateral PTC patients, especially those with multifocal disease. By comprehensively assessing the individual risk of contralateral OTC and the presence of CLNM ≥5, we stratified patients into distinct risk groups. Tailored surgical treatments based on this stratification were then given to prevent overtreatment in low-risk patients while mitigating recurrence risk in high-risk individuals, thereby enabling individualized surgical management. Additionally, leveraging postoperative pathological data from patients who had undergone TL, we constructed a pathological model to predict the risk of contralateral OTC. This model was designed to guide postoperative management for the TL patients, including TSH suppression intensity and surveillance frequency.

## Methods

### Patient selection

This study collected clinical data from patients who had undergone TT at Union Hospital, Tongji Medical College, Huazhong University of Science and Technology, Wuhan, China, from May 2020 to May 2024.

Study participants were enrolled according to the following inclusion and exclusion criteria. Inclusion Criteria: Complete medical records; Preoperative ultrasonographically-suspected malignant nodule(s)(unifocal or multifocal) in a unilateral thyroid lobe (left/right), with FNAB cytopathology classified as Bethesda Category V or higher; Initial surgery consisting of TT plus central lymph node dissection (CLND); Pathological diagnosis of PTC; No history of neck irradiation. Exclusion Criteria: Incomplete medical records; Preoperative ultrasonography indicating suspicious malignant nodule(s) at bilateral thyroid lobes or isthmus, with malignancy confirmed by FNAB; Initial surgery consisting of TL ± CLND; Other TC pathological subtypes (*e.*g., oncocytic carcinoma, follicular carcinoma, medullary thyroid carcinoma, anaplastic thyroid carcinoma); History of neck irradiation.

**Figure 1:**
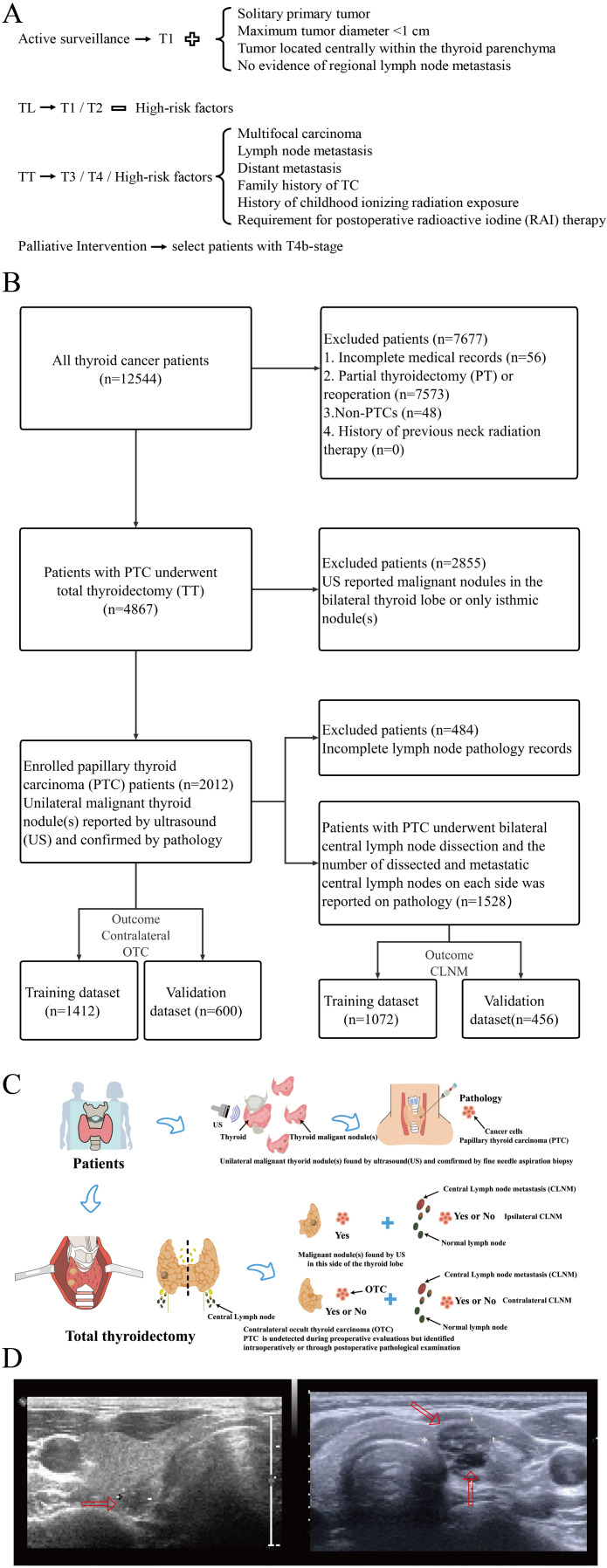
**(A)** Surgical decision-making per Chinese guidelines adopted in our research center; **(B)** Patient selection flowchart; **(C)** Schematic diagram of study design; **(D)** Representative ultrasonographic images (Left: unilateral unifocal PTC; right: unilateral multifocal PTC).

Patients were initially identified when their preoperative ultrasonography exclusively suspected malignant nodule(s) in a unilateral lobe (unifocal/multifocal). Upon confirmation of PTC by FNAB, patients having undergone initial TT were further selected. **Figure 1A** illustrates the surgical decision-making process followed at our center, which is based on the Chinese guidelines. Those without prior neck irradiation were included for clinicopathological examinations, with contralateral OTC as a primary endpoint. For the secondary endpoint of CLNM number, patients undergoing bilateral CLND with adequate lymph node harvest were subjected to subsequent analyses. **Figures 1B, C** present the patient selection flowchart and the study design schematic, respectively.

This study was approved by the Institutional Review Board of Wuhan Union Hospital, Tongji Medical College, Huazhong University of Science and Technology (Approval No [2025]. 0004).

### Data collection and variable selection

Patient medical record data were obtained from the electronic medical record system, including basic information (age, gender, and body mass index [BMI]), ultrasonographic features (the number and the SLD of unilateral malignant nodules, presence of malignant isthmus nodules, calcification, anteroposterior-to-transverse ratio, capsular disruption, malignant nodules located near the isthmus), thyroid function (hyperthyroidism, hypothyroidism, chronic lymphocytic thyroiditis [CLT]), pathological features (pathological subtype, number of cancer foci, SLD of cancer foci, intraglandular dissemination, ETE, isthmus cancer, nodular goiter, vascular invasion, neural invasion, and CLNM). Clinical characteristics including demographics, ultrasonographic findings, and thyroid function were preoperatively available. Representative ultrasonographic images are shown in **Figure 1D**.

### Data analysis

Statistical analyses were performed using SPSS version 7.00 (Chicago, IL, USA), R version 4.2.2 (The R Foundation for Statistical Computing), and Python version 3.9.12. Categorical variables were analyzed using the chi-square test, and continuous variables were compared using the independent samples *t*-test. A *p*-value <0.05 indicated statistically significant difference. Patients were randomly allocated to training and validation cohorts in a 7:3 ratio, ensuring no significant intergroup differences (p>0.05). Variance inflation factor (VIF) was calculated to exclude multicollinearity.

To develop clinical prediction models with optimal precision and reliability, seven machine learning algorithms were employed: Decision Tree (DT); Elastic Net Regression (ENet); Logistic Regression; Multilayer Perceptron (MLP); Random Forest (RF); Support Vector Machine (SVM); eXtreme Gradient Boosting (XGBoost). A five-fold cross-validation framework was implemented for resampling. Optimal hyperparameter combinations were identified via grid search within the cross-validation scheme to train final models. Model performance was evaluated using relevant metrics, including the area under the receiver operating characteristic curve (AUC), recall, sensitivity, and specificity. Discriminative ability, calibration, and clinical utility were further assessed using receiver operating characteristic (ROC) curves (20), calibration curves (21), and decision curve analysis (DCA) (22), respectively. The optimal model was selected based on a comprehensive evaluation. The logistic regression model demonstrated superior performance. Variables with *p*<0.05 in univariate logistic regression were entered into multivariate analysis. Significant predictors (*p*<0.05) from the multivariate analysis were incorporated into the final risk prediction model. A nomogram was constructed for model visualization. Additionally, SHapley Additive exPlanations (SHAP) (23) analysis was performed to quantify feature importance and interpret model predictions by calculating each feature’s contribution to the outcome.

For pathological model development, 12 machine learning algorithms and 113 algorithm combinations were evaluated: Lasso Regression; Ridge Regression; ENet; Stepwise Generalized Linear Model (Stepglm); SVM; Gradient Boosting with Component-wise Linear Models (glmBoost); Linear Discriminant Analysis (LDA); Partial Least Squares Regression generalized linear models (plsRglm); RF; Gradient Boosting Machines (GBM); XGBoost; Naive Bayes Classifier. One algorithm selected features, while the other one built the predictive model within the cross-validation framework. AUC values were calculated for all combinations in both training and validation cohorts. Model performance was visualized via a heatmap, and the combination achieving the highest mean AUC was employed to construct the final pathological model.

## Results

### Clinical prediction models of contralateral OTC and CLNM were established on the basis of the variables available before surgery

#### Construction and validation of the contralateral OTC prediction model

##### Clinical characteristics of patients

A total of 12,544 patients with TC were initially identified through the electronic medical record system. Against the inclusion and exclusion criteria, 10,532 patients were excluded, resulting in a final cohort of 2,012 patients for analysis. Within this cohort, 572 patients (28.43%) had pathologically confirmed contralateral OTC. Clinical characteristics of the study population are detailed in **Table 1**.

**Table 1:** Patient baseline characteristics in the clinical prediction model for contralateral OTC.

Characteristics	OTC n=572^1^	Non-OTC n=1440^1^	Statistic	p
BMI (kg/m^2^)				
Mean ± SD	24.0 ± 3.4	23.1 ± 3.2	5.13	<0.001^4^
<24	313 (54.7%)	941 (65.3%)	27.93	<0.001^2^
24≤BMI<28	179 (31.3%)	390 (27.1%)		
≥28	80 (14.0%)	109 (7.6%)		
Age				
Mean ± SD	42 ± 12	43 ± 12	-1.87	0.062^4^
≤55	491 (85.8%)	1 208 (83.9%)	1.19	0.276^2^
>55	81 (14.2%)	232 (16.1%)		
Gender			12.94	<0.001^3^
Female	410 (71.7%)	1 143 (79.4%)		
Male	162 (28.3%)	297 (20.7%)		
Hyperthyroidism			8.18	0.004^2^
No	566 (99.0%)	1 392 (96.7%)		
Yes	6 (1.0%)	48 (3.3%)		
Hypothyroidism				0.115^3^
No	572 (100.0%)	1 432 (99.4%)		
Yes	0 (0.0%)	8 (0.6%)		
CLT			4.93	0.026^2^
No	366 (64.0%)	844 (58.6%)		
Yes	206 (36.0%)	596 (41.4%)		
Number				
Mean ± SD	1.47 ± 0.72	1.13 ± 0.39	10.53	<0.001^4^
1	365 (63.8%)	1 274 (88.5%)	164.86	<0.001^2^
>1	207 (36.2%)	166 (11.5%)		
SLD (cm)				
Mean ± SD	1.83 ± 1.19	1.19 ± 0.74	12.08	<0.001^4^
≤1	153 (26.7%)	793 (55.1%)	188.85	<0.001^2^
1<SLD ≤ 2	251 (43.9%)	488 (33.9%)		
2<SLD ≤ 4	144 (25.2%)	157 (10.9%)		
>4	24 (4.2%)	2 (0.1%)		
Calcification			3.05	0.081^2^
No	131 (22.9%)	384 (26.7%)		
Yes	441 (77.1%)	1 056 (73.3%)		
Anteroposterior-to-transverse ratio			3.08	0.079^2^
<1	310 (54.2%)	718 (49.9%)		
≥1	262 (45.8%)	722 (50.1%)		
Malignant nodules located near the isthmus			0.69	0.405^2^
No	530 (92.7%)	1 349 (93.7%)		
Yes	42 (7.3%)	91 (6.3%)		
Malignant isthmus nodule(s)			103.44	<0.001^2^
No	508 (88.8%)	1 422 (98.8%)		
Yes	64 (11.2%)	18 (1.3%)		
Capsular disruption			49.11	<0.001^2^
No	119 (20.8%)	533 (37.0%)		
Yes	453 (79.2%)	907 (63.0%)		

^1^n (%), ^2^Pearson’s Chi-squared test, ^3^Fisher’s exact test, ^4^Welch Two Sample t-test.

##### Independent variables screening and prediction model development

**Figure 2:**
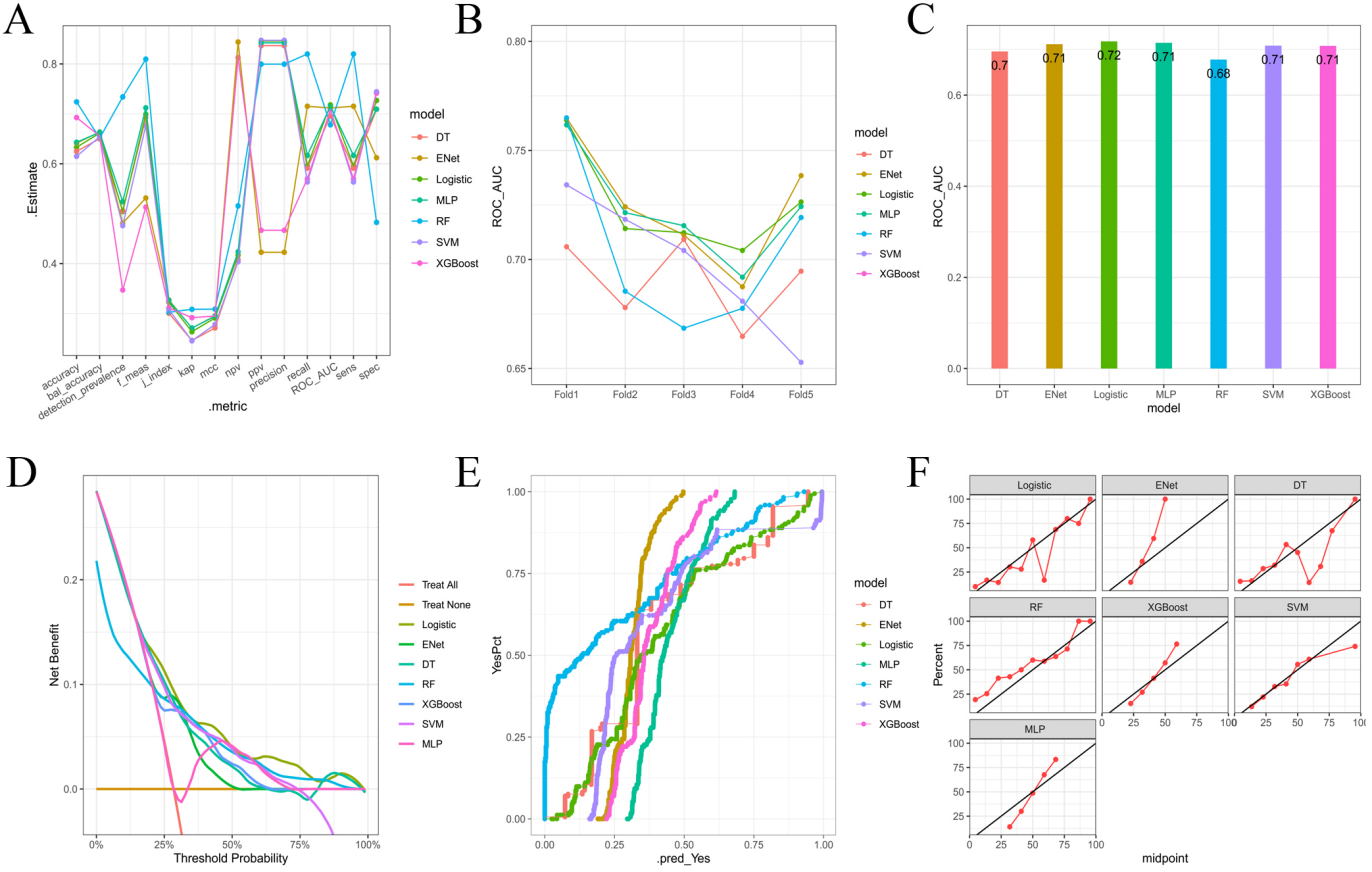
**(A)** Parallel lines of error indexes of each clinical model on the validation cohort; **(B)** ROC-AUC index of each model in different fold; **(C)** ROC-AUC index of each model on the validation cohort; **(D)** DCA curves for each model on the validation cohort; **(E, F)** Calibration curve of each model on the validation cohort.

Multiple machine learning algorithm models were constructed and evaluated using key performance metrics, including recall, accuracy, precision, and specificity (**Figure 2**). The logistic regression model consistently demonstrated superior performance across all parameters without extreme outliers. Univariate and multivariate logistic regression analyses (**Table 2**) identified five independent predictors: BMI; isthmic malignant nodule; number of malignant nodules within unilateral lobe; SLD of malignant nodules; capsular disruption. All VIFs were <10, confirming the absence of multicollinearity. A point-based scoring system was developed using regression coefficients from multivariate analysis to facilitate clinical risk stratification.

**Table 2:** Univariate and multivariable regression analysis for clinical factors associated with contralateral OTC.

Characteristics	Univariate analysis			Multivariate analysis				
	OR	95%CI	p	OR	95%CI	p	b	Fast score
BMI (kg/m²)								
<24	1	(reference)		1	(reference)			0
≤24<BMI<28	1.40	1.07∼1.83	0.014	1.33	0.99∼1.81	0.063	0.29	1
≥28	2.34	1.61∼3.39	<0.001	1.81	1.18∼2.77	0.007	0.59	2
Gender								
Female	1	(reference)		1	(reference)			
Male	1.45	1.11∼1.89	0.007	1.12	0.82∼1.54	0.465		
Number								
1	1	(reference)		1	(reference)			0
>1	4.13	3.14∼5.45	<0.001	2.41	1.73∼3.36	<0.001	0.88	3
SLD (cm)								
≤1	1	(reference)		1	(reference)			0
1<SLD ≤ 2	2.96	2.23∼3.92	<0.001	1.95	1.42∼2.68	<0.001	0.67	2
2<SLD ≤ 4	4.94	3.51∼6.96	<0.001	2.94	1.97∼4.39	<0.001	1.08	4
>4	40.77	9.12∼182.22	<0.001	13.86	2.91∼65.96	<0.001	2.63	9
Malignant isthmus nodule(s)								
No	1	(reference)		1	(reference)			0
Yes	10.06	5.47∼18.51	<0.001	5.51	2.78∼10.92	<0.001	1.71	6
Capsular disruption								
No	1	(reference)		1	(reference)			0
Yes	2.68	2.01∼3.57	<0.001	2.15	1.56∼2.95	<0.001	0.76	3

**Figure 3:**
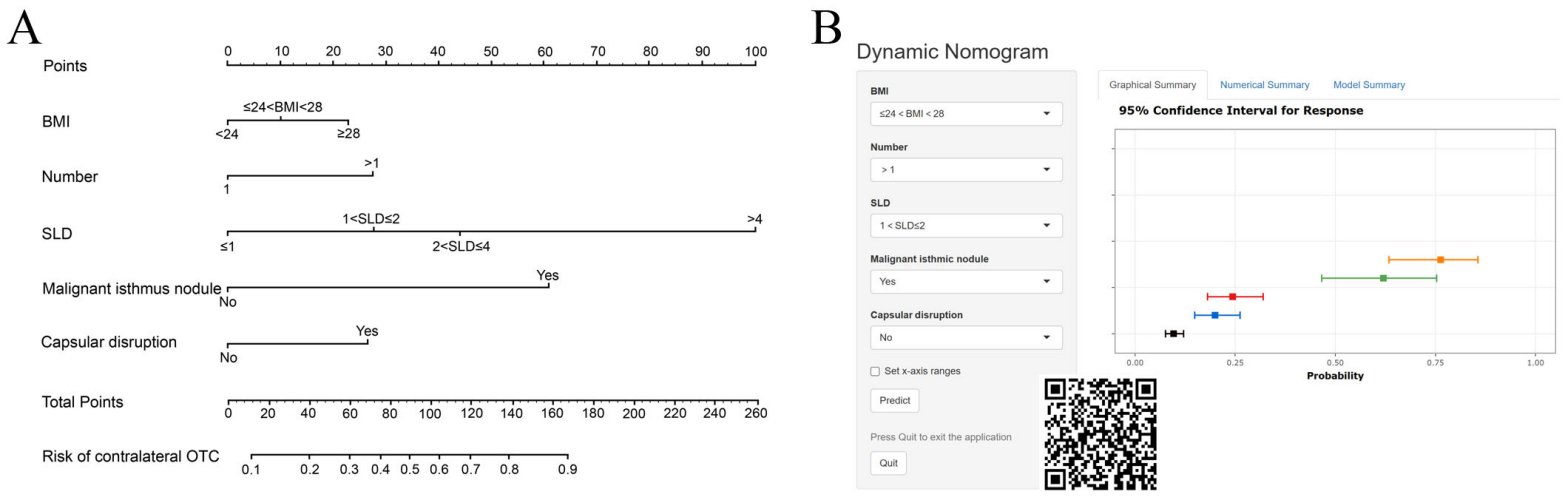
Nomogram **(A)** and dynamic nomogram **(B)** of prediction models for contralateral OTC.

The selected variables were integrated into the final risk prediction model. A nomogram graphically representing the model is shown in **Figure 3A**. To enhance patient accessibility, we developed an interactive web-based nomogram (https://model1.shinyapps.io/dynnomapp/). Patients may access this tool via web browser or QR code scanning. By selecting their corresponding variable categories, users could obtain real-time estimates of their individualized outcome probability (**Figure 3B**).

**Figure 4:**
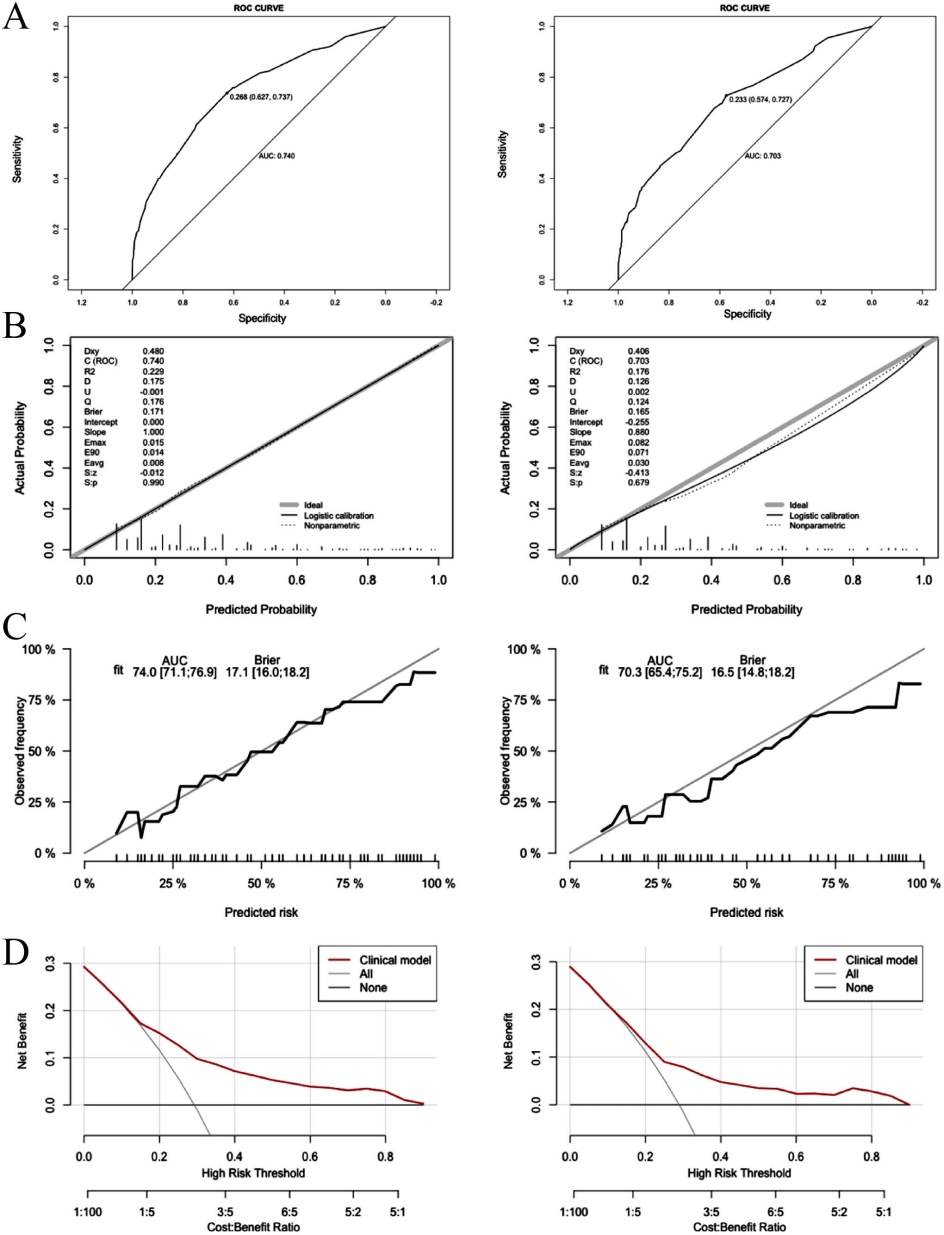
Logistic model for contralateral OTC (Left: training cohort; right: validation cohort). **(A)** ROC curves of internal validation in clinical prediction model; **(B, C)** Calibration curves of internal validation in clinical prediction model; **(D)** DCA curves of internal validation in clinical prediction model.

The discriminatory performance of the clinical prediction model was evaluated using ROC curves, yielding an AUC values of 0.740 in the training cohort and an AUC of 0.703 in the validation cohort (**Figure 4A**). Calibration was assessed via the Hosmer-Lemeshow goodness-of-fit test, which indicated good agreement between predicted and observed outcomes (training cohort: χ² = 7.60, *p* = 0.58; validation cohort: χ² = 11.47, *p* = 0.25). This calibration performance was further visualized through calibration curves (**Figures 4B, C**). Clinical utility was evaluated using DCA curves. The model demonstrated significantly greater net benefit than both “treat-none” and “treat-all” strategies across threshold probabilities ranging from 10% to 90%, indicating superior clinical applicability for guiding intervention-related decision-making (**Figure 4D**).

### Model interpretation with SHAP

**Figure 5:**
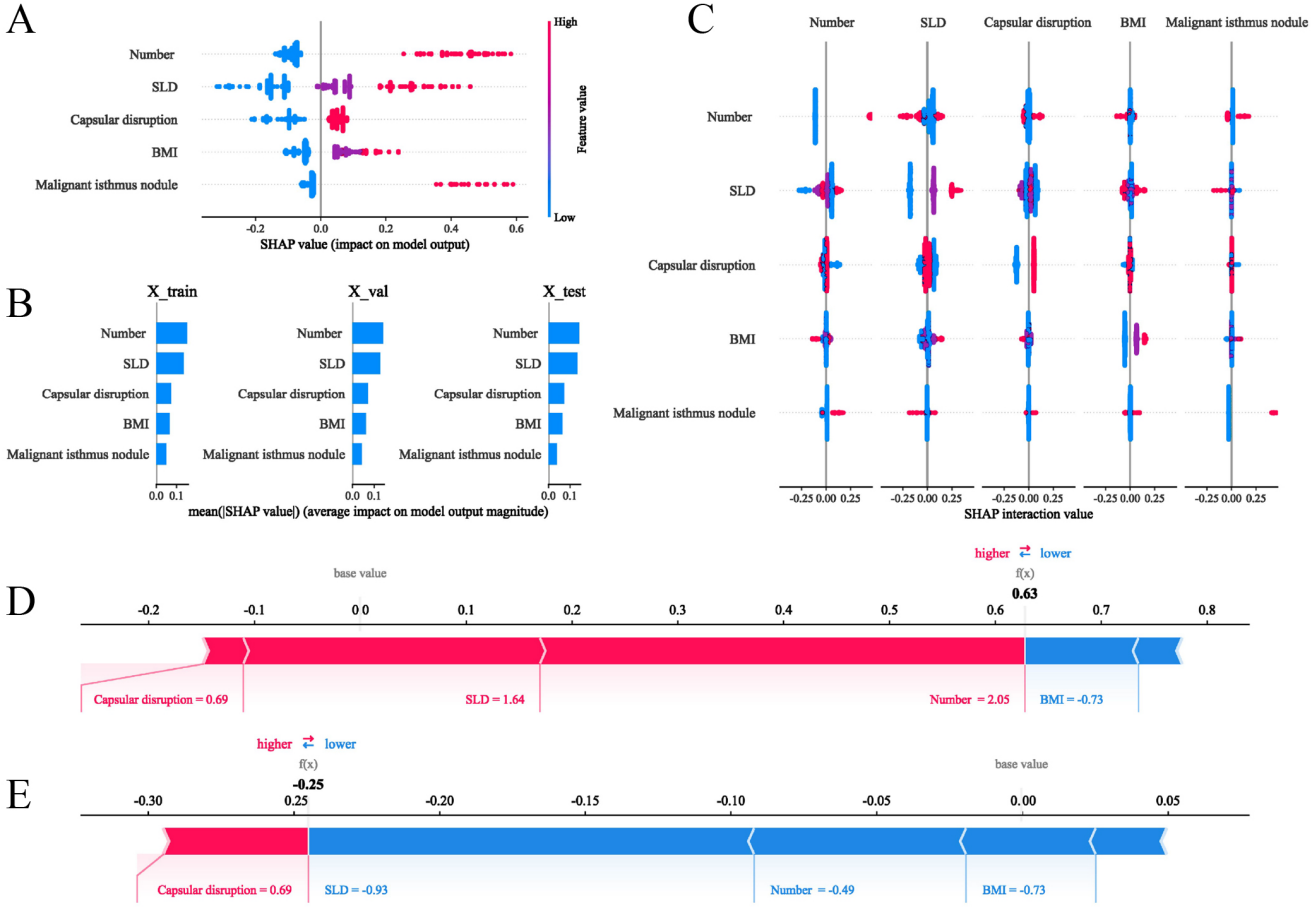
SHAP interpretations of the clinical model for the prediction of the contralateral OTC. **(A)** SHAP values of five features in the model; **(B)** Ranking of features’ average absolute SHAP values. The matrix describes the importance of each covariate in the development of the final predictive model; **(C)** The importance and impact of interactions between features. Features are ranked by importance. The total importance value of each feature is the sum of the importance values of its interactions with other features. Each point represents the interaction value of a sample, and the color represents the feature interacting with the main feature; **(D, E)** show how SHAP values explain the predicted contralateral OTC probabilities in two individuals.

To provide intuitive interpretation of selected variables, SHapley Additive exPlanations (SHAP) visualization was employed. This method quantifies each feature’s contribution to model predictions, explaining how variables influence the risk of contralateral OTC in the clinical model. **Figure 5A** displays the five predictor variables. Each dot represents a sample’s feature value for model outputting, with red indicating positive contributions (increased OTC risk) and blue denoting negative contributions (decreased risk). Multifocality, larger SLD, capsular disruption, higher BMI, and presence of isthmic malignant nodules were associated with elevated contralateral OTC risk. **Figure 5B** ranks feature importance in terms of mean SHAP values. Features at the top exerted the most significant influence when their values varied. **Figure 5C** visualizes interaction effects between features. The total importance value represents the sum of interaction magnitudes across feature pairs. Each point corresponds to a sample’s interaction value, and color gradients denote interaction directions. Additionally, two representative cases further demonstrated model interpretability. The length and direction of bars reflect the contribution level of each feature. **Figure 5D** shows that patient with contralateral OTC gave a high SHAP value (0.63). Conversely, a patient without contralateral OTC yielded a negative prediction score (-0.25) (**Figure 5E**).

#### Construction and validation of the CLNM (≥ 5) prediction model

##### Baseline characteristics of patients

A cohort of 1,528 patients who underwent TT with bilateral CLND was identified through the medical record system. Among them, 542 (35.47%) had CLNM ≥5. Baseline clinical characteristics of the study population are summarized in **Table 3**.

**Table 3:** Patient baseline characteristics in the prediction model for CLNM (≥ 5).

Characteristics	≥5 CLNM n=542^1^	<5 CLNM n=986^1^	Statistic	p
BMI (kg/m^2^)				
Mean ± SD	23.7 ± 3.6	23.4 ± 3.3	1.30	0.195^2^
<24	307 (56.6%)	603 (61.2%)	12.87	0.002^3^
≤24<BMI<28	159 (29.3%)	302 (30.6%)		
≥28	76 (14.0%)	81 (8.2%)		
Age				
Mean ± SD	38 ± 11	45 ± 11	-12.21	<0.001^2^
≤55	496 (91.5%)	792 (80.3%)	33.07	<0.001^3^
>55	46 (8.5%)	194 (19.7%)		
Gender			60.22	<0.001^3^
Female	349 (64.4%)	810 (82.2%)		
Male	193 (35.6%)	176 (17.8%)		
Hyperthyroidism			5.09	0.024^3^
No	534 (98.5%)	952 (96.6%)		
Yes	8 (1.5%)	34 (3.4%)		
Hypothyroidism				0.661^4^
No	541 (99.8%)	982 (99.6%)		
Yes	1 (0.2%)	4 (0.4%)		
CLT			20.77	<0.001^3^
No	356 (65.7%)	529 (53.7%)		
Yes	186 (34.3%)	457 (46.3%)		
Number			5.51	0.019^3^
1	362 (66.8%)	715 (72.5%)		
>1	180 (33.2%)	271 (27.5%)		
SLD (cm)				
Mean ± SD	1.56 ± 1.01	1.01 ± 0.76	11.06	<0.001^2^
≤1	168 (31.0%)	608 (61.7%)	160.39	<0.001^3^
1<SLD ≤ 2	263 (48.5%)	324 (32.9%)		
2<SLD ≤ 4	97 (17.9%)	49 (5.0%)		
>4	14 (2.6%)	5 (0.5%)		
Calcification			44.60	<0.001^3^
No	75 (13.8%)	286 (29.0%)		
Yes	467 (86.2%)	700 (71.0%)		
Anteroposterior-to-transverse ratio			17.28	<0.001^3^
<1	312 (57.6%)	458 (46.5%)		
≥1	230 (42.4%)	528 (53.5%)		
Capsular disruption			35.92	<0.001^3^
No	105 (19.4%)	334 (33.9%)		
Yes	437 (80.6%)	652 (66.1%)		
Malignant nodules located near the isthmus			8.78	0.003^3^
No	487 (89.9%)	927 (94.0%)		
Yes	55 (10.1%)	59 (6.0%)		
Malignant isthmus nodule(s)			7.17	0.007^3^
No	505 (93.2%)	949 (96.2%)		
Yes	37 (6.8%)	37 (3.8%)		

^1^n (%), ^2^Welch Two Sample t-test, ^3^Pearson’s Chi-squared test, ^4^Fisher’s exact test.

##### Independent variable screening and prediction model development

**Figure 6:**
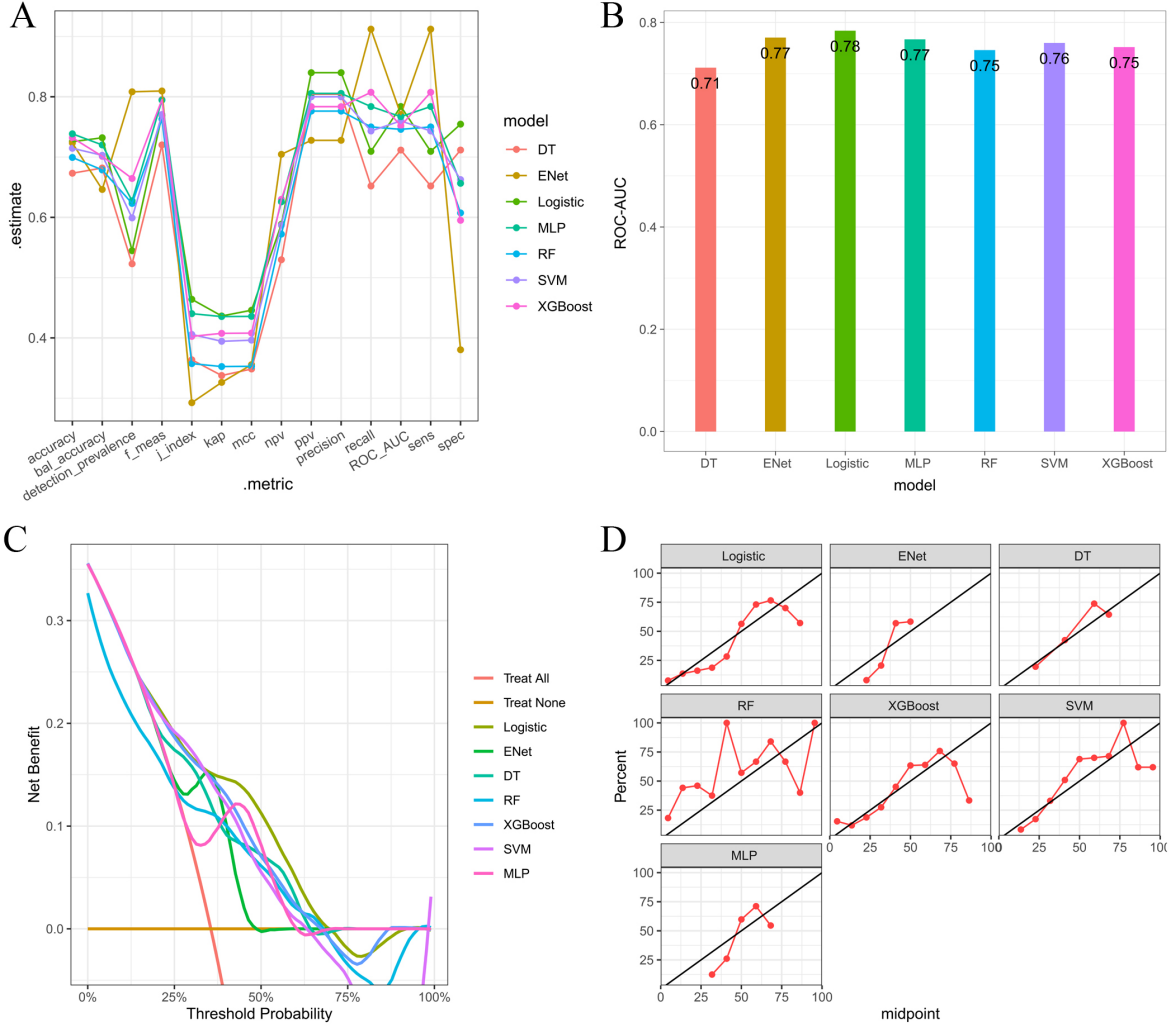
**(A)** Parallel lines of error indexes of each clinical model on the validation cohort; **(B)** ROC-AUC index of each model on the validation cohort; **(C)** DCA curves for each model on the validation cohort; **(D)** Calibration curves of each model on the validation cohort.

After a comprehensive evaluation of recall, accuracy, precision, specificity, discrimination, calibration, and clinical utility, the logistic regression model was selected as the primary model for the prediction of CLNM risk (**Figure 6**, **Table 4**).

**Table 4:** Univariate and multivariable regression analysis for clinical factors associated with CLNM (≥5).

Characteristics	Univariate analysis			Multivariate analysis				
	OR	95%CI	p	OR	95%CI	p	b	Fast score
Age								
>55	1	(reference)		1	(reference)			0
≤55	2.38	1.63∼3.47	<0.001	2.27	1.51∼3.43	<0.001	0.82	3
Gender								
Female	1	(reference)		1	(reference)			0
Male	2.50	1.88∼3.33	<0.001	2.23	1.62∼3.05	<0.001	0.80	3
Calcification								
No	1	(reference)		1	(reference)			0
Yes	2.52	1.81∼3.50	<0.001	2.13	1.49∼3.04	<0.001	0.75	2
Malignant nodules located near the isthmus								
No	1	(reference)		1	(reference)			0
Yes	1.78	1.11∼2.84	0.016	2.01	1.19∼3.41	0.009	0.70	2
CLT								
Yes	1	(reference)		1	(reference)			0
No	1.62	1.25∼2.10	<0.001	1.38	1.04∼1.84	0.027	0.32	1
SLD (cm)								
≤1	1	(reference)		1	(reference)			0
1<SLD ≤ 2	2.70	2.04∼3.56	<0.001	1.95	1.44∼2.64	<0.001	0.67	2
2<SLD ≤ 4	5.80	3.66∼9.20	<0.001	3.68	2.24∼6.05	<0.001	1.30	4
>4	13.32	3.70∼47.95	<0.001	8.11	2.09∼31.54	0.003	2.67	8
Capsular disruption								
No	1	(reference)		1	(reference)			0
Yes	2.63	1.94∼3.57	<0.001	1.95	1.39∼2.73	<0.001	0.67	2

**Figure 7:**
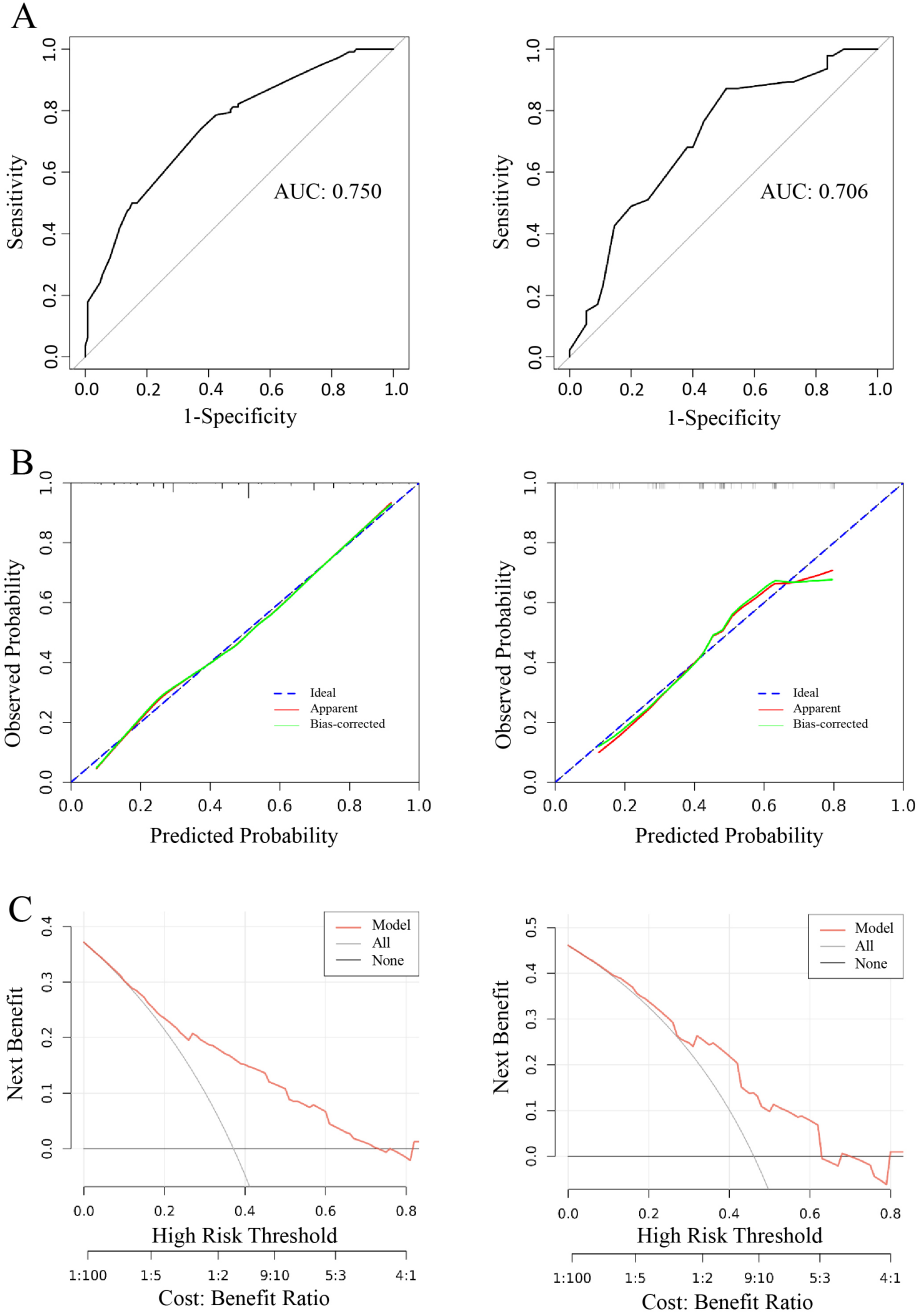
Logistic model for CLNM≥5 (Left: training cohort; right: validation cohort). **(A)** ROC curve of internal validation in clinical prediction model; **(B)** Calibration curves of internal validation in clinical prediction model; **(C)** DCA curves of internal validation in clinical prediction model.

The predictive model demonstrated a robust performance, with an AUC of 0.750 (training cohort) and 0.706 (validation cohort), respectively (**Figure 7A**). Calibration curves indicated an excellent agreement between predicted probabilities and observed outcomes, as supported by Hosmer-Lemeshow goodness-of-fit test results (*p* > 0.05 for both cohorts) (**Figure 7B**). DCA curves revealed that model-guided interventions provided net benefit over other strategies at threshold probabilities of 10%-70% in PTC patients (**Figure 7C**).

**Figure 8:**
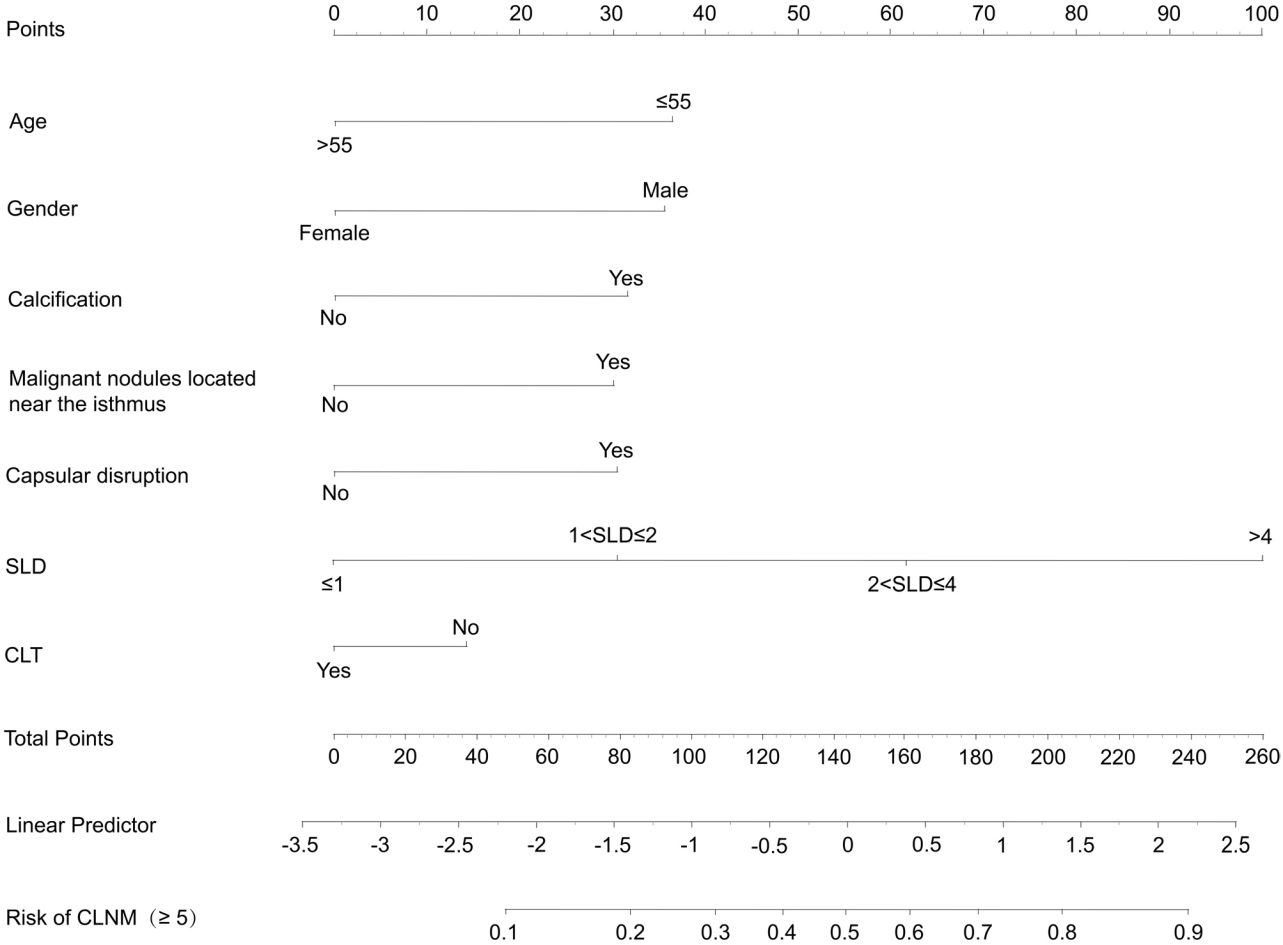
Nomogram of prediction models for CLNM≥5.

The final clinical nomogram (**Figure 8**) integrated ultrasonographic predictors, including malignant nodules near the isthmus, SLD, capsular disruption, and calcifications, with clinical variables (age, sex, CLT) to stratify risk of ≥5 CLNM.

### Correlation between contralateral OTC and CLNM

Spearman correlation analysis revealed a statistically significant association between contralateral OTC and CLNM (*p* < 0.05). Patients with contralateral OTC demonstrated significantly higher rates of CLNM (≥5) compared to their counterparts without OTC (*p* < 0.05, **Table 5**).

**Table 5:** Chi-square test to assess the association between contralateral OTC and CLNM (≥5).

Characteristic	≥5 CLNM n=542^1^	<5 CLNM n=986^1^	χ^2^	p
Contralateral OTC				
No	294 (54.2%)	717 (72.7%)	53.32	<0.001
Yes	248 (45.8%)	269 (27.3%)		

^1^n (%).

### The risk stratification of the predictive model and the corresponding fast score

**Figure 9:**
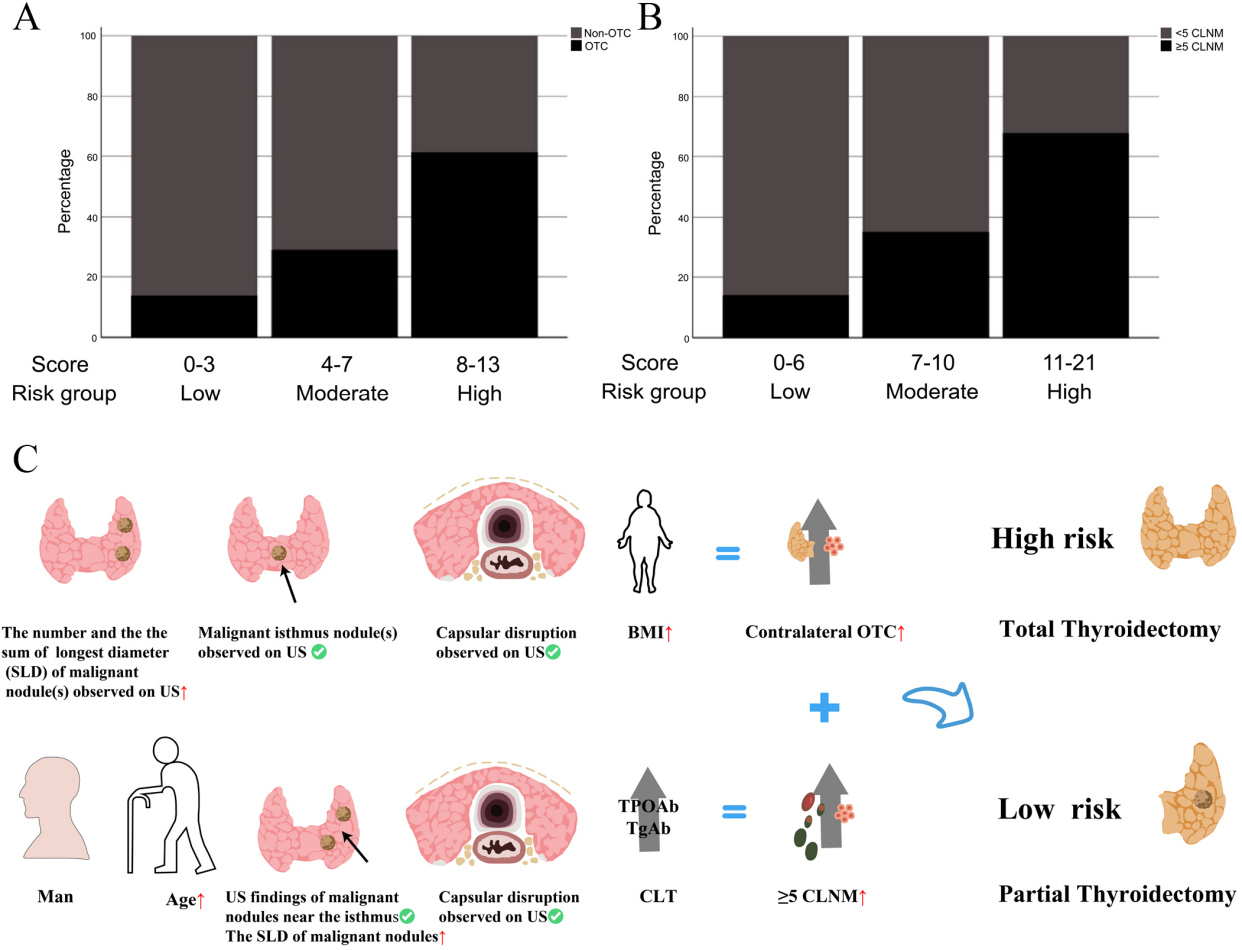
**(A, B)** The incidences of contralateral OTC and CLNM (≥5) in different risk groups stratified by fast scores in the clinical model; **(C)** Schematic diagram of surgical treatment selection.

Patients were re-stratified and categorized into distinct risk tiers based on cumulative risk scores derived from the predictive models. Stacked bar plots demonstrated a progressively increasing proportion of positive outcomes with higher cumulative scores (**Figures 9A, B**). Utilizing the OTC and CLNM risk stratification system, we proposed tailored surgical strategies for treatment-naïve PTC patients: High-risk patients should receive TT to mitigate recurrence, while low-risk patients are indicated for TL to prevent overtreatment. The decision-making with those at intermediate risk needs to incorporate quantified risk probabilities and patients’ choice (**Figure 9C**).

### A contralateral OTC clinical prediction model was made by using postoperative variables to provide guidance for postoperative follow-up of PT patients who had undergone PT

**Figure 10:**
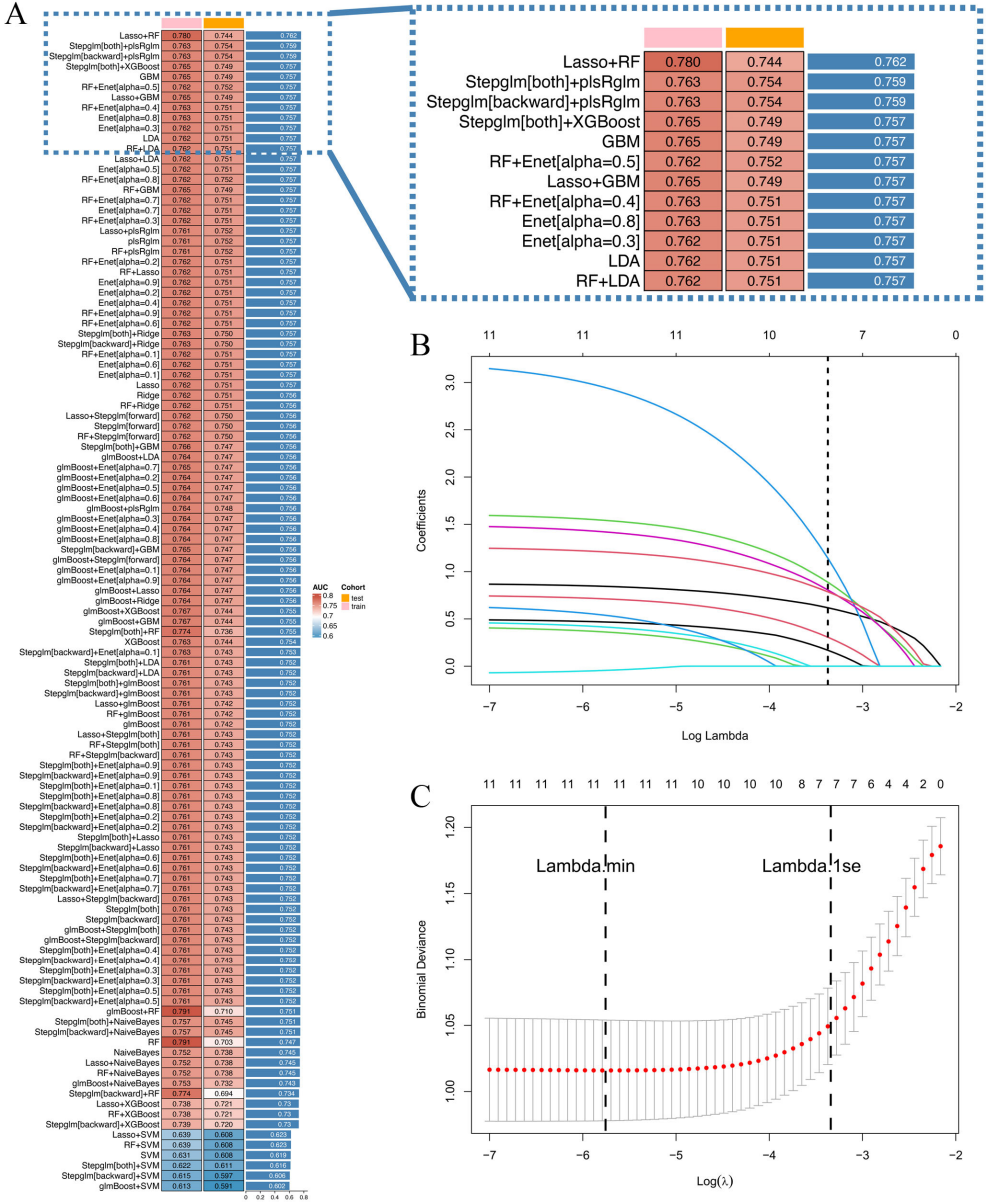
**(A)** 12 machine learning algorithms were integrated, resulting in 113 algorithm combinations, and the AUC index of each pathological model was calculated; **(B)** Lasso regression variable selection trajectory; **(C)** Cross-validation error curve for Lasso regression.

**Figure 11:**
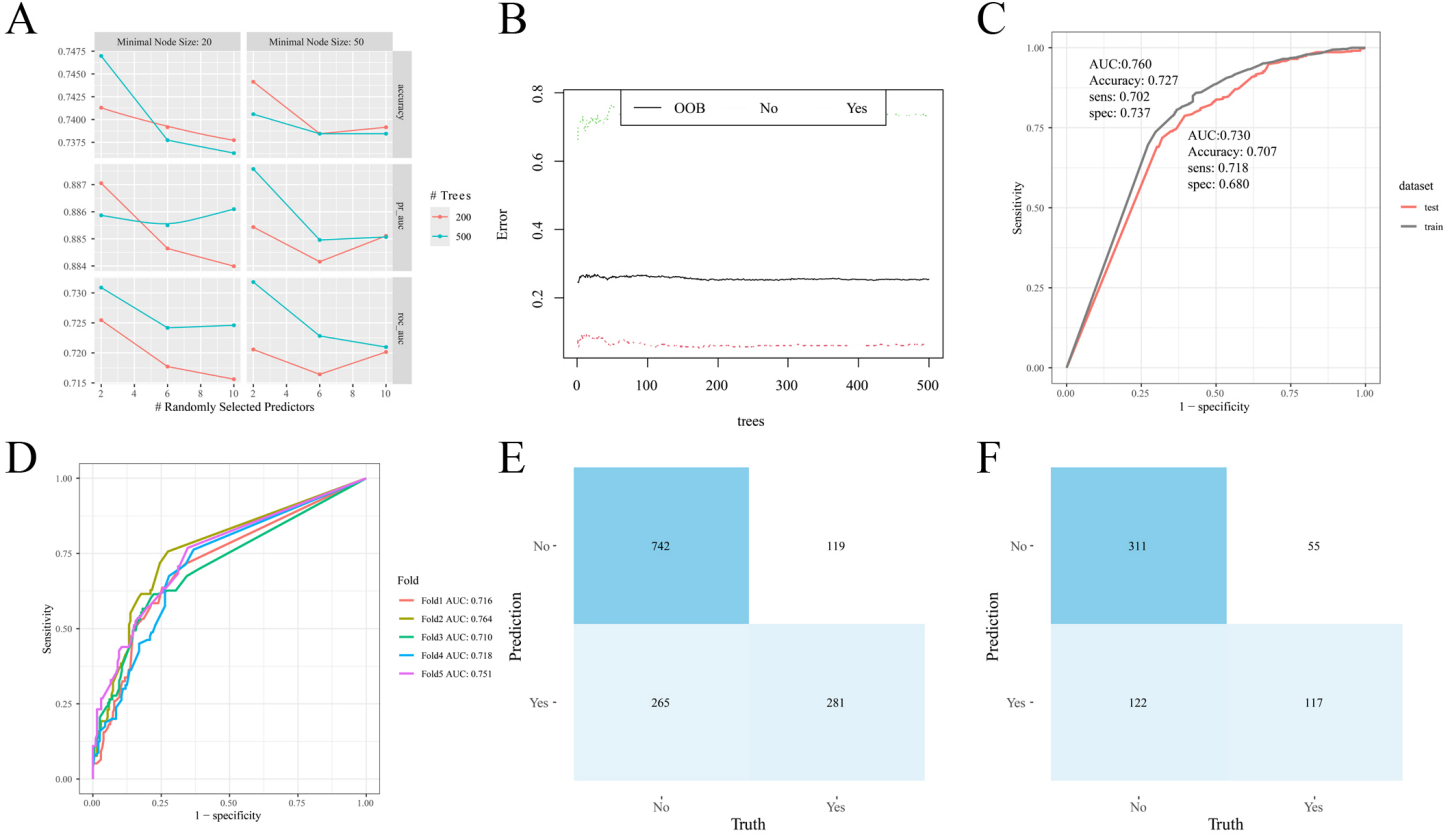
Random forest model construction and validation. **(A)** Cross-validation results; **(B)** Number and error of random forest trees; **(C)** ROC curve of the model on training and validation cohorts; **(D)** ROC curve of the model in different folds; **(E)** Confusion matrix (training cohort); **(F)** Confusion matrix (validation cohort).

**Figure 12:**
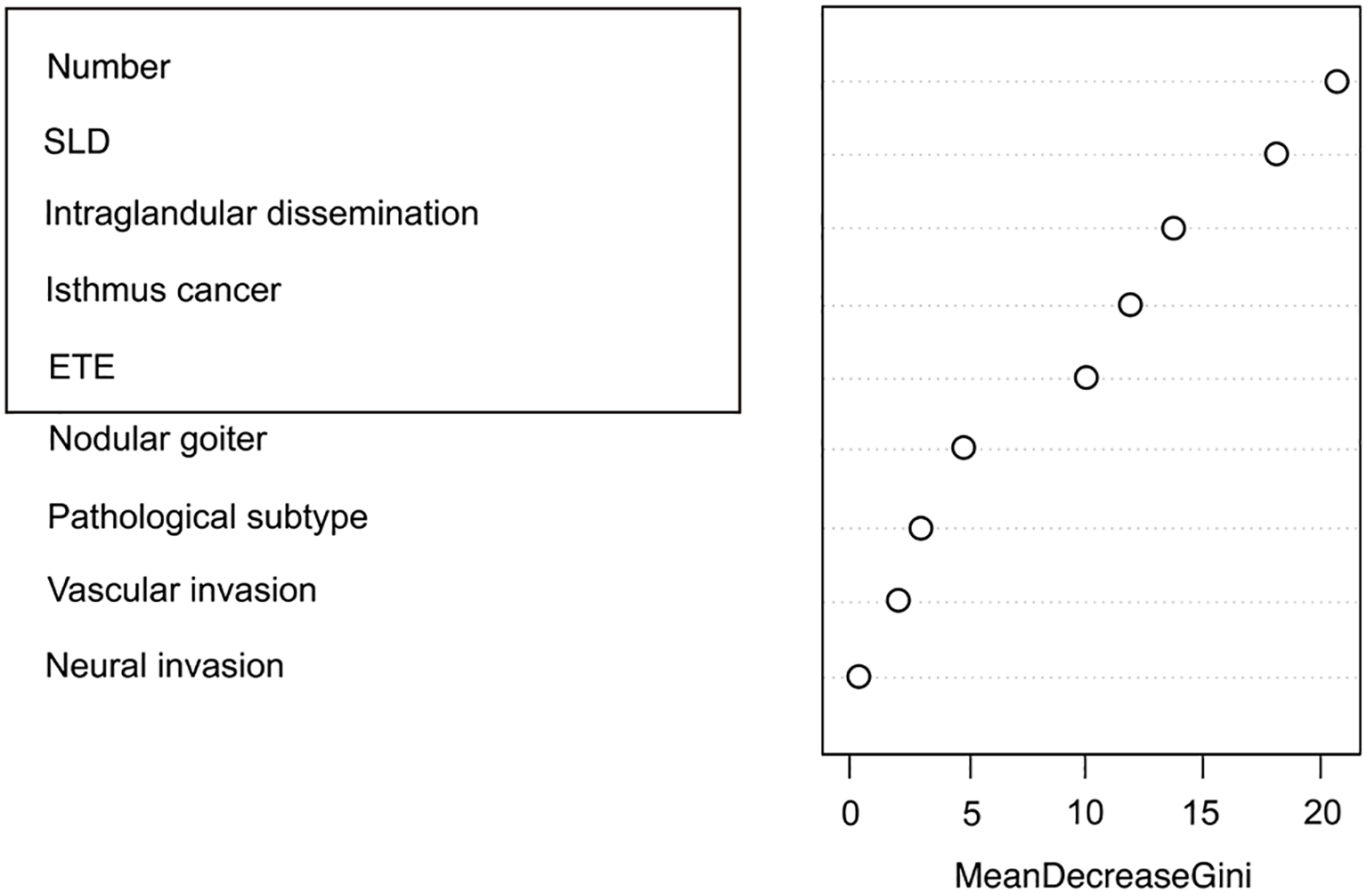
Ranking of pathological feature importance for contralateral OTC prediction (Random forest algorithm).

Clinicopathological characteristics of the 2,012 enrolled patients are detailed in **Table 6**. A heatmap analysis of AUC indices across 12 machine learning algorithms and 113 combinations revealed that the Lasso + RF ensemble achieved the highest mean AUC (0.762) (**Figure 10A**). At lambda.1se, Lasso regression identified five pathological features with non-zero coefficients: number of pathologically-confirmed cancer foci, SLD of cancer foci, isthmic cancer, intraglandular dissemination, and ETE (**Figures 10B, C**). Subsequent RF model using these predictors employed optimal hyperparameters (mtry = 2, trees = 500, min_n = 50) determined through cross-validation (**Figure 11A**). **Figure 11B** illustrates the relationship between the Random Forest model's error and the number of decision trees. The model demonstrated robust discriminative power (training AUC: 0.760; validation AUC: 0.730; **Figure 11C**) and stable performance on five-fold cross-validation (mean AUC 0.732 ± 0.011 SE; **Figure 11D**). **Figure 11E** and **11F** present the confusion matrices for the training and validation cohorts, respectively. Feature importance analysis in terms of mean decrease in Gini impurity further established that the contribution level of predictors was in the following order: number > SLD > intraglandular dissemination > isthmic cancer > ETE (**Figure 12**).

**Table 6:** Patient baseline characteristics in the pathological prediction model for contralateral OTC.

Characteristics	OTC n=572^1^	Non-OTC n=1440^1^	Statistic	p
Number of nodule(s) on pathology				
Mean ± SD	1.65 ± 0.94	1.22 ± 0.49	10.54	<0.001^3^
1	323 (56.5%)	1 176 (81.7%)	136.84	<0.001^2^
>1	249 (43.5%)	264 (18.3%)		
SLD on pathology (cm)				
Mean ± SD	1.60 ± 1.14	0.93 ± 0.61	13.32	<0.001^3^
≤1	200 (35.0%)	939 (65.2%)	203.73	<0.001^2^
1<SLD ≤ 2	258 (45.1%)	429 (29.8%)		
2<SLD ≤ 4	94 (16.4%)	70 (4.9%)		
>4	20 (3.5%)	2 (0.1%)		
Isthmus cancer on pathology			103.44	<0.001^2^
No	508 (88.8%)	1 422 (98.8%)		
Yes	64 (11.2%)	18 (1.3%)		
Pathological subtype			3.07	0.080^2^
Classic	478 (83.6%)	1 247 (86.6%)		
Non-Classic	94 (16.4%)	193 (13.4%)		
Intraglandular dissemination			90.51	<0.001^2^
No	474 (82.9%)	1 377 (95.6%)		
Yes	98 (17.1%)	63 (4.4%)		
Vascular invasion			9.87	0.002^2^
No	546 (95.5%)	1 411 (98.0%)		
Yes	26 (4.5%)	29 (2.0%)		
Neural invasion			2.76	0.097^2^
No	558 (97.6%)	1 420 (98.6%)		
Yes	14 (2.4%)	20 (1.4%)		
ETE			48.34	<0.001^2^
No	494 (86.4%)	1 372 (95.3%)		
Yes	78 (13.6%)	68 (4.7%)		
Nodular goiter			9.69	0.002^2^
No	374 (65.4%)	833 (57.8%)		
Yes	198 (34.6%)	607 (42.2%)		

^1^n (%), ^2^Pearson’s Chi-squared test, ^3^Welch Two Sample t-test.

OTC, occult thyroid carcinoma; ETE, extrathyroidal extension; SLD, the sum of longest diameter; CLNM, central lymph node metastasis; CLT, chronic lymphocytic thyroiditis.

## Discussion

In 2022, TC emerged as the third most prevalent malignancy in China, with 466,100 new cases diagnosed, making the condition a major oncological burden (24). With the evidence-based medicine evolving, surgical paradigms for PTC have undergone substantial refinement: from initial tumor-focused excision to radical resection that underscores recurrence prevention, and now toward an individualized strategy that aims to achieve an optimal balance between oncological control and functional preservation. Most TC cases are subclinical PTC, which is typically asymptomatic, behaviorally indolent and has a stable disease-specific mortality over a period of three decades (2). Consequently, the major challenge confronting PTC management lies in avoiding undertreatment of high-risk patients while preventing overtreatment of low-risk individuals. Current Chinese Guidelines for the Diagnosis and Treatment of Thyroid Nodules and Differentiated Thyroid Cancer, alongside American Thyroid Association (ATA) recommendations are major yardsticks that guide our clinical decision-making. However, controversies remain regarding the optimal surgical approach for unilateral PTCs, especially for multifocal cases. TT lowers locoregional recurrence risk, thereby minimizing the probability of reoperation and facilitating postoperative radioactive iodine (RAI) therapy (25). This choice must be weighed against elevated risks for surgical complications, including transient or permanent hypoparathyroidism and recurrent laryngeal nerve injury (26), alongside the necessity for lifelong high-dose thyroid hormone replacement (27). Conversely, advocates of TL emphasize its advantages in functional preservation of parathyroid, laryngeal nerve and residual thyroid, though at an acceptable price of marginally higher risk of recurrence in the remnant lobe (28).

This study developed predictive models for contralateral OTC and CLNM to inform surgical decision-making. The pathological analysis revealed two critical findings, including CLNM burden and contralateral OTC. 35.47% of the patients (542/1528) had ≥5 CLNM, exceeding ATA high-risk threshold and 28.43% (572/2012) harbored occult carcinomas. Crucially, 76.9% (440/572) of these OTCs measured <2 mm and were ultrasonographically undetectable. OTC is histopathologically confirmable thyroid malignancies that are undetectable during preoperative evaluations but can be identified intraoperatively or postoperatively, with an incidence rate spanning from 13% to 56% reported in TT or near-TT specimens (29). Current evidence demonstrated that OTC exhibited a biological aggressiveness comparable to macroscopic tumors (30), with a regional nodal metastasis rate of up to 33% (31) and a potential of hematogenous dissemination. The bilaterally interwoven lymphatic network of the thyroid facilitates sequential tumor spread from the central compartment to the contralateral lobe and lateral neck nodes and is mechanistically culpable for the strong association between CLNM and contralateral OTC (meta-analysis OR = 2.086, 95% CI = 1.246–3.495, p = 0.005). Particularly, Delphian node metastasis demonstrates high specificity in predicting contralateral OTC, potentially justifying conversion to total thyroidectomy when intraoperative frozen section yields positive results. CLNM status stratified OTC risk effectively, with 61.8% (21/34) of CLNM-positive patients harboring contralateral OTC against a mere 4.5% (56/385) in their CLNM-negative counterparts. Current preoperative diagnostics remain inadequate, since ultrasound and computer tomography (CT) achieve an sensitivity of only 28.4% and 40.0%, respectively, for CLNM detection (32), while magnetic resonance imaging (MRI) and central node biopsy are not readily accessible. Consequently, our models integrate preoperative predictors to quantitatively evaluate individualized OTC/CLNM risks, providing validated thresholds to inform decision-making about surgical extent while striking a balance between oncological control and functional preservation.

In our cohort of patients undergoing TT+CLND, 732 exhibited neither OTC nor >5 CLNM on final pathology, indicating that a substantial proportion could have attained functional preservation of thyroid by avoiding total resection. After model-based recalibration, 384 patients were re-classified as candidates for initial TL. Current guidelines base surgical decisions on TNM staging, high-risk profile, and nodal status, while our approach leverages preoperative clinical factors through a dual-outcome model that assesses contralateral OTC and CLNM risks. Analysis identified a distinct risk profile: elevated BMI, larger SLD, multifocality, capsular disruption, or isthmic malignant nodules indicative of higher OTC probability; younger age, male sex, absence of CLT, ultrasonographic features, including malignant nodules near the isthmus, larger SLD, capsular disruption, and calcification suggestive of extensive CLNM (≥5). For unilateral PTC, particularly multifocal disease, our model provides personalized guidance. Patients with multiple high-risk features undergoing TL exhibited significantly elevated probability of requiring reoperation for contralateral recurrence, suggesting that when extensive CLNM coexists, the heightened contralateral risk justifies TT despite its inherent risks, as the potential benefits in reducing reoperation outweigh the risk of complications. On the other hand, TL remains preferable for unifocal microcarcinomas given its lower recurrence risk and superior functional preservation. Consequently, we recommend TL for model-defined low-risk patients to prevent overtreatment and advocate TT for high-risk individuals to mitigate recurrence. This method overcomes the critical limitation of preoperative unavailability of key pathological determinants (margin status, vascular invasion, extensive CLNM) that traditionally guide preoperative decisions.

Compared to prior predictive models for surgical decision-making concerning PTC, this study innovatively utilized exclusively preoperative indicators to provide guidance before operation on resection extent, offering distinct advantages over traditional approaches depending on postoperative pathology. We further employed machine learning methodologies to identify optimal algorithms through comprehensive parameter evaluation and found that SHAP analysis could enhance model interpretability and clinical applicability (33). By simultaneously assessing risks of contralateral OTC and ≥5 CLNM, our approach delivers holistic evidence for surgical planning, complemented by a point-based scoring system allowing for rapid preoperative risk stratification. Notwithstanding these advances, this study is subject to limitations inherent with retrospective design, including potential selection heterogeneity and biases during data collection. Future prospective validation is essential to refining these models, and large-size multicenter (multinational) external validation is still warranted to arrive at definitive conclusions.

Additionally, the study exclusively included patients with Bethesda Category V-VI nodules (malignancy risk: 67-83%), whereas the revised 2023 TBSRTC third edition now recommends diagnostic TL for Category III/IV nodules. This shift carries significant clinical implications, particularly for Category III, which is atypia of undetermined significance (AUS; malignancy risk 13-30%) (34). Evaluating indeterminate AUS cases is still one of the most challenging issues (35), decisions about active surveillance or diagnostic surgery should integrate molecular testing, clinical risk factors (family history, radiation exposure), sonographic features (36), and patients’ choice. The appropriate application of FANB is essential to minimize the risk of overlooking thyroid malignancies (37). Consequently, extending this study to Category III nodules represents a critical next step, with future studies poised to develop tailored surgical strategy for this diagnostically challenging subgroup.

This study developed and validated clinical prediction models for contralateral OTC and CLNM based on logistic regression-identified risk factors. Analyses revealed that patients with elevated BMI, larger SLD, multifocality, capsular disruption, or isthmic malignant nodules had heightened susceptibility to contralateral OTC. Conversely, younger age, male sex, absence of CLT, ultrasonographic features (malignant nodules near the isthmus, larger SLD, capsular disruption), and calcification were predictive of increased risk of extensive CLNM (≥5). Risk-stratified treatment strategies for treatment-naïve PTC patients were established on the basis of OTC and CLNM model scores: When both scores fall within the high-risk ranges (OTC: 8–13 points; CLNM: 11–21 points), TT is recommended. For low-risk patients (OTC: 0–3 points; CLNM: 0–6 points), TL is advised to mitigate overtreatment risks, to specifically prevent permanent hypothyroidism and minimize potential injury to parathyroid glands and recurrent laryngeal nerves. These models provide clinically actionable insights for precision surgery, thereby pushing forward personalized management of unilateral PTC.

## Data Availability

The original contributions presented in the study are included in the article/supplementary material. Further inquiries can be directed to the corresponding author.

## Glossary

AUC: Area under the receiver operating characteristic curve
ATA: American Thyroid Association
BMI: Body mass index
CLNM: Central lymph node metastasis
CLT: Chronic lymphocytic thyroiditis
CT: Computer tomography
DCA: Decision curve analysis
DT: Decision Tree
DTC: Differentiated thyroid cancer
ENet: Elastic NetEnet
ETE: Extrathyroidal extension
GBM: Gradient Boosting Machines
glmBoost: Gradient Boosting with Component-wise Linear Models
HL: Hosmer-Lemeshow
LDA: Linear Discriminant Analysis
MLP: Multilayer Perceptron
MRI: Magnetic resonance imaging
OTC: Occult thyroid carcinoma
plsRglm: Partial Least Squares Regression generalized linear models
PTC: Papillary thyroid carcinoma
RF: Random Forest
ROC: Receiver operating characteristic
SHAP: SHapley Additive exPlanations
SLD: Sum of longest diameter
Stepglm: Stepwise generalized linear model
SVM: Support vector machines
TC: Thyroid cancer
TL: Thyroid lobectomy
TT: Total thyroidectomy
VIF: Variance inflation factor
XGBoost: EXtremeGradientBoosting

## References

[1] Pacini F Castagna MG Brilli L Pentheroudakis G. Thyroid cancer: ESMO Clinical Practice Guidelines for diagnosis, treatment and follow-up. Ann Oncol. (2012) 23 Suppl 7:vii110–119. doi: 10.1093/annonc/mds230, PMID: 22997443

[2] Sung H Ferlay J Siegel RL Laversanne M Soerjomataram I Jemal A. Global cancer statistics 2020: GLOBOCAN estimates of incidence and mortality worldwide for 36 cancers in 185 countries. CA: A Cancer J Clin. (2021) 71:209–49. doi: 10.3322/caac.21660, PMID: 33538338

[3] McLeod DSA Zhang L Durante C Cooper DS. Contemporary debates in adult papillary thyroid cancer management. Endocrine Rev. (2019) 40:1481–99. doi: 10.1210/er.2019-00085, PMID: 31322698

[4] Homayouni M Mohammad Arabzadeh SA Nili F Razi F Amoli MM. Evaluation of the presence of Epstein-Barr virus (EBV) in Iranian patients with thyroid papillary carcinoma. Pathol Res Pract. (2017) 213:854–6. doi: 10.1016/j.prp.2017.01.020, PMID: 28554750

[5] de Almeida JFM Ward LS. Thyroid autoimmune diseases and thyroid tumors: Would EBV infection be the link? J Cell Physiol. (2019) 234:19141–2. doi: 10.1002/jcp.28641, PMID: 31120135

[6] Germano A Schmitt W Almeida P Mateus-Marques R Leite V. Ultrasound requested by general practitioners or for symptoms unrelated to the thyroid gland may explain higher prevalence of thyroid nodules in females. Clin Imaging. (2018) 50:289–93. doi: 10.1016/j.clinimag.2018.05.003, PMID: 29738997

[7] Boucai L Zafereo M Cabanillas ME. Thyroid cancer: A review. JAMA. (2024) 331:425–35. doi: 10.1001/jama.2023.26348, PMID: 38319329

[8] Callender GG Carling T Christison-Lagay E Udelsman R. Surgery for thyroid cancer. Endocrinol Metab Clinics North America. (2014) 43:443–58. doi: 10.1016/j.ecl.2014.02.011, PMID: 24891171

[9] Dal Maso L Vaccarella S Franceschi S. Trends in thyroid cancer incidence and overdiagnosis in the USA. Lancet Diabetes Endocrinol. (2025) 13:167–9. doi: 10.1016/S2213-8587(24)00343-7, PMID: 39922211

[10] Haddad RI Bischoff L Ball D Bernet V Blomain E Busaidy NL. Thyroid carcinoma, version 2.2022, NCCN clinical practice guidelines in oncology. J Natl Compr Cancer Network. (2022) 20:925–51. doi: 10.6004/jnccn.2022.0040, PMID: 35948029

[11] Harries V Wang LY McGill M Xu B Tuttle RM Wong RJ. Should multifocality be an indication for completion thyroidectomy in papillary thyroid carcinoma? Surgery. (2020) 167:10–7. doi: 10.1016/j.surg.2019.03.031, PMID: 31515125 PMC6904525

[12] Cabanillas ME McFadden DG Durante C. Thyroid cancer. Lancet. (2016) 388:2783–95. doi: 10.1016/S0140-6736(16)30172-6, PMID: 27240885

[13] Haugen BR Alexander EK Bible KC Doherty GM Mandel SJ Nikiforov YE. 2015 American thyroid association management guidelines for adult patients with thyroid nodules and differentiated thyroid cancer: the American thyroid association guidelines task force on thyroid nodules and differentiated thyroid cancer. Thyroid. (2016) 26:1–133. doi: 10.1089/thy.2015.0020, PMID: 26462967 PMC4739132

[14] Scharpf J Tuttle M Wong R Ridge D Smith R Hartl D. Comprehensive management of recurrent thyroid cancer: An American Head and Neck Society consensus statement: AHNS consensus statement. Head Neck. (2016) 38:1862–9. doi: 10.1002/hed.24513, PMID: 27717219

[15] Tufano RP Clayman G Heller KS Inabnet WB Kebebew E Shaha A. Management of recurrent/persistent nodal disease in patients with differentiated thyroid cancer: a critical review of the risks and benefits of surgical intervention versus active surveillance. Thyroid. (2015) 25:15–27. doi: 10.1089/thy.2014.0098, PMID: 25246079

[16] Lu WW Zhang D Ni XJ. A review of the role of ultrasound radiomics and its application and limitations in the investigation of thyroid disease. Med Sci Monitor. (2022) 28:e937738. doi: 10.12659/MSM.937738, PMID: 36258648 PMC9587688

[17] Zhao H Li H. Meta-analysis of ultrasound for cervical lymph nodes in papillary thyroid cancer: Diagnosis of central and lateral compartment nodal metastases. Eur J Radiol. (2019) 112:14–21. doi: 10.1016/j.ejrad.2019.01.006, PMID: 30777203

[18] Hartl DM Guerlain J Breuskin I Hadoux J Baudin E Al Ghuzlan A. Thyroid lobectomy for low to intermediate risk differentiated thyroid cancer. Cancers. (2020) 12(11):3282. doi: 10.3390/cancers12113282, PMID: 33171949 PMC7694652

[19] Kluijfhout WP Pasternak JD Lim J Kwon JS Vriens MR Clark OH. Frequency of high-risk characteristics requiring total thyroidectomy for 1-4 cm well-differentiated thyroid cancer. Thyroid. (2016) 26:820–4. doi: 10.1089/thy.2015.0495, PMID: 27083216

[20] Nahm FS. Receiver operating characteristic curve: overview and practical use for clinicians. Korean J Anesthesiol. (2022) 75:25–36. doi: 10.4097/kja.21209, PMID: 35124947 PMC8831439

[21] Cheng WL Markus C Lim CY Tan RZ Sethi SK Loh TP. Calibration practices in clinical mass spectrometry: review and recommendations. Ann Lab Med. (2023) 43:5–18. doi: 10.3343/alm.2023.43.1.5, PMID: 36045052 PMC9467832

[22] Vickers AJ Holland F. Decision curve analysis to evaluate the clinical benefit of prediction models. Spine J. (2021) 21:1643–8. doi: 10.1016/j.spinee.2021.02.024, PMID: 33676020 PMC8413398

[23] Lundberg SM Erion G Chen H DeGrave A Prutkin JM Nair B. From local explanations to global understanding with explainable AI for trees. Nat Mach Intell. (2020) 2:56–67. doi: 10.1038/s42256-019-0138-9, PMID: 32607472 PMC7326367

[24] Han B Zheng R Zeng H Wang S Sun K Chen R. Cancer incidence and mortality in China, 2022. Zhonghua Zhong Liu Za Zhi Chin J Oncol. (2024) 46:221–31. doi: 10.1016/j.jncc.2024.01.006, PMID: 38468501

[25] Padur AA Kumar N Guru A Badagabettu SN Shanthakumar SR Virupakshamurthy MB. Safety and effectiveness of total thyroidectomy and its comparison with subtotal thyroidectomy and other thyroid surgeries: A systematic review. J Thyroid Res. (2016) 2016:7594615. doi: 10.1155/2016/7594615, PMID: 27006857 PMC4783568

[26] Abood A Ovesen T Rolighed L Triponez F Vestergaard P. Hypoparathyroidism following total thyroidectomy: high rates at a low-volume, non-parathyroid institution. Front Endocrinol. (2024) 15:1330524. doi: 10.3389/fendo.2024.1330524, PMID: 38304463 PMC10833226

[27] Xue S Wang P Liu J Chen G. Total thyroidectomy may be more reasonable as initial surgery in unilateral multifocal papillary thyroid microcarcinoma: a single-center experience. World J Surg Oncol. (2017) 15:62. doi: 10.1186/s12957-017-1130-7, PMID: 28302162 PMC5356282

[28] Hsiao V Light TJ Adil AA Tao M Chiu AS Hitchcock M. Complication rates of total thyroidectomy vs hemithyroidectomy for treatment of papillary thyroid microcarcinoma: A systematic review and meta-analysis. JAMA Otolaryngol Head Neck Surg. (2022) 148:531–9. doi: 10.1001/jamaoto.2022.0621, PMID: 35511129 PMC9073663

[29] Koo BS Lim HS Lim YC Yoon YH Kim YM Park YH. Occult contralateral carcinoma in patients with unilateral papillary thyroid microcarcinoma. Ann Surg Oncol. (2010) 17:1101–5. doi: 10.1245/s10434-009-0906-6, PMID: 20066517

[30] Piana S Ragazzi M Tallini G de Biase D Ciarrocchi A Frasoldati A. Papillary thyroid microcarcinoma with fatal outcome: evidence of tumor progression in lymph node metastases: report of 3 cases, with morphological and molecular analysis. Hum Pathol. (2013) 44:556–65. doi: 10.1016/j.humpath.2012.06.019, PMID: 23079204

[31] Martinez-Tello FJ Martinez-Cabruja R Fernandez-Martin J Lasso-Oria C Ballestin-Carcavilla C. Occult carcinoma of the thyroid. A systematic autopsy study from Spain of two series performed with two different methods. Cancer. (1993) 71:4022–9. doi: 10.1002/1097-0142(19930615)71:12<4022::AID-CNCR2820711236>3.0.CO;2-O, PMID: 8508367

[32] Xing Z Qiu Y Yang Q Yu Y Liu J Fei Y. Thyroid cancer neck lymph nodes metastasis: Meta-analysis of US and CT diagnosis. Eur J Radiol. (2020) 129:109103. doi: 10.1016/j.ejrad.2020.109103, PMID: 32574937

[33] Sengul I Sengul D. Reinterpretation of a new ChatGPT-empowered, easy-to-use machine learning paradigm: An aide-memoire. Eco Environ Health. (2025) 4:100156. doi: 10.1016/j.eehl.2025.100156, PMID: 40521087 PMC12166422

[34] Sengul I Sengul D. Blurred lines for management of thyroid nodules in the era of atypia of undetermined significance/follicular lesion of undetermined significance: novel subdivisions of categories IIIA and IIIB in a possible forthcoming The Bethesda System for Reporting Thyroid Cytopathology, 3rd edition; amending versus unnecessary? Rev Assoc Med Bras (1992). (2021) 67:1385–6. doi: 10.1590/1806-9282.20210763, PMID: 35018962

[35] Sengul I Sengul D. Evangely, the subcategorization has been announced in the 2023 Bethesda system for reporting thyroid cytopathology: let bygones be bygones in thyroidology! Rev Assoc Med Bras (1992). (2024) 70:e20231511. doi: 10.1590/1806-9282.20231511, PMID: 38656016 PMC11042832

[36] Sengul I Sengul D. Focusing on thyroid nodules in suspense: 10–15 mm with repeat cytology, Category III, the Bethesda System for Reporting Thyroid Cytopathology, TBSRTC. Rev Assoc Med Bras (1992). (2021) 67:166–7. doi: 10.1590/1806-9282.67.02.20200828, PMID: 34406237

[37] Sengul D Sengul I. Subdivision of intermediate suspicion, the 2021 K-TIRADS, and category III, indeterminate cytology, the 2017 TBSRTC, 2nd edition, in thyroidology: let bygones be bygones? Ultrasonography. (2023) 42:600–1. doi: 10.14366/usg.23113, PMID: 37691418 PMC10555690

